# Prognostic Implications of Biventricular Global Longitudinal Strain in Patients With Severe Isolated Tricuspid Regurgitation

**DOI:** 10.3389/fcvm.2022.908062

**Published:** 2022-08-03

**Authors:** Dae-Young Kim, Jiwon Seo, Iksung Cho, Seung Hyun Lee, Sak Lee, Geu-Ru Hong, Jong-Won Ha, Chi Young Shim

**Affiliations:** ^1^Department of Cardiology, CHA Bundang Medical Center, CHA University School of Medicine, Seongnam-si, South Korea; ^2^Division of Cardiology, Severance Cardiovascular Hospital, Yonsei University College of Medicine, Seoul, South Korea; ^3^Division of Cardiovascular Surgery, Department of Thoracic and Cardiovascular Surgery, Severance Cardiovascular Hospital, Yonsei University College of Medicine, Seoul, South Korea

**Keywords:** isolated tricuspid regurgitation, global longitudinal strain, left ventricle, right ventricle, surgery, prognosis

## Abstract

**Background:**

Isolated TV surgery can be performed in patients with symptoms caused by severe isolated tricuspid regurgitation (TR), preferably before the onset of significant right ventricular (RV) dysfunction. In patients with severe TR, intrinsic RV dysfunction tends to be masked and promotes left ventricular (LV) mechanical dysfunction. This study investigated the prognostic implications of biventricular global longitudinal strain (GLS) in patients receiving isolated tricuspid valve (TV) surgery.

**Methods:**

Among 1,670 patients who underwent TV surgery between January 2000 and December 2020, 111 patients with severe isolated TR who underwent echocardiography before and after TV surgery were analyzed. We assessed LV, RV, and biventricular GLS using speckle tracking echocardiography. Biventricular GLS was defined as the sum of LV-GLS and RV free-wall strain. The primary outcomes were cardiovascular death, heart failure hospitalization, re-done TV surgery, and heart transplantation.

**Results:**

During 3.9 ± 3.8 years of follow-up after the postoperative echocardiography, 24 (21.6%) patients experienced a primary outcome. Those patients had more comorbidities and more impaired preoperative RV-GLS and biventricular GLS than those who did not experience a primary outcome, although the two groups did not differ in preoperative LV-GLS. Patients with a primary outcome also showed significantly impaired postoperative RV-GLS, biventricular GLS, and LV-GLS compared those without a primary outcome. In multivariate analyses, both pre- and postoperatively assessed RV-GLS [preoperative; hazard ratio (HR) 0.86, confidence interval (CI) 0.79–0.93, *p* < 0.001, postoperative; HR 0.89, CI 0.82–0.96, *p* = 0.004] and biventricular GLS [preoperative; HR 0.96, CI 0.91–1.00, *p* = 0.048, postoperative; HR 0.94, CI 0.89–0.99, *p* = 0.023] were independently associated with the primary outcomes.

**Conclusion:**

In patients with severe isolated TR undergoing TV surgery, the absolute value of RV-GLS under 17.2% is closely associated with a poor prognosis, and that of biventricular GLS under 34.0%, mainly depending on the RV-GLS, is related to the poor prognosis. Further prospective multicenter studies are warranted to establish the risk stratification of isolated TV surgery.

## Introduction

Isolated tricuspid regurgitation (TR), which is not associated with left-sided heart disease or pulmonary hypertension, has received increased attention because it correlates with early mortality even without any cardiovascular comorbidities (1, 2). It has been recognized that isolated tricuspid valve (TV) surgery could offer prognostic benefit before the development of significant right ventricular (RV) dysfunction or end-organ failure. However, current practice guidelines do not present clear surgical timing for isolated TV surgery due to limited and controversial study results (3, 4).

Most patients with severe isolated TR have chronic RV volume overload, which leads to deteriorating RV function unless intervention is timely (5). Moreover, severe isolated TR can mask intrinsic RV dysfunction. Ultimately, RV dilation and dysfunction promote left ventricular (LV) under-filling, which produces mechanical LV dysfunction (6). Therefore, a comprehensive assessment of both LV and RV function in patients with severe isolated TR is important in predicting prognostic outcomes after isolated TV surgery. However, using echocardiography to evaluate LV and RV function in patients with severe isolated TR is challenging due to the complex anatomic structure of the RV chamber and under-filling of the LV chamber (7). Two-dimensional speckle-tracking echocardiography is more sensitive and less volume-dependent in assessing LV and RV systolic function than conventional transthoracic echocardiography (8–11). However, few reports have used biventricular global longitudinal strain (GLS) to predict the clinical outcomes of patients with severe isolated TR. Therefore, we hypothesized that pre- and postoperative biventricular GLS would provide prognostic information for patients with severe isolated TR.

## Materials and Methods

### Study Population

**FIGURE 1:**
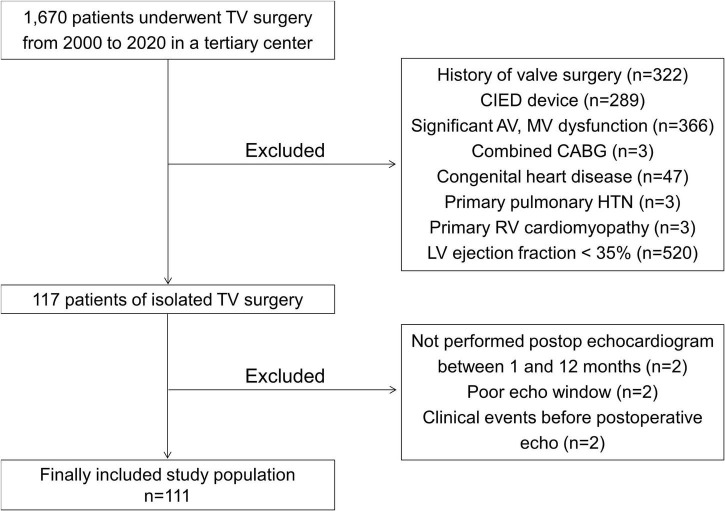
A flowchart of this study. TV, tricuspid valve; CIED, cardiovascular implantable electronic devices; AV, aortic valve; MV, mitral valve; CABG, coronary artery bypass graft; HTN, hypertension; RV, right ventricle; LV, left ventricle.

We retrospectively identified 1,670 patients who underwent TV surgery (TV repair or replacement) in a single tertiary center between January 2000 and December 2020. Among them, we excluded patients who had a history of prior TV, aortic valve, or mitral valve surgery, had a cardiovascular implantable electronic device, had concomitant significant (at least moderate) aortic or mitral valve dysfunction, had combined coronary artery bypass surgery, had congenital heart disease, had been diagnosed with pulmonary hypertension (pulmonary artery systolic pressure greater than 50 mmHg), had a primary RV cardiomyopathy such as arrhythmogenic cardiomyopathy, or had an LV ejection fraction (EF) of less than 35%. We also excluded patients who did not undergo preoperative transthoracic echocardiography within 6 months before isolated TV surgery and postoperative echocardiography between 1 and 12 months after isolated TV surgery and those whose echocardiographic images were too poor to analyze ventricular GLS. All patients who had clinical events between TV surgery and the day of postoperative echocardiography were also excluded. After those exclusions, we included 111 patients in this study population (Figure 1). Each patient’s clinical history, medications, laboratory results, echocardiographic parameters, and clinical outcomes were reviewed retrospectively. The etiology of isolated TR was classified as follows: the primary TR, which included the defect of TV leaflet itself, and the secondary TR, which was related to tricuspid annular dilation with normal leaflet morphology. This study was conducted in accordance with the Declaration of Helsinki, and the study protocol was approved by the Institutional Review Board of Yonsei University Health System (IRB number: 4-2021-0929).

### Follow-Up and Outcomes

After isolated TV surgery, the patients visited the outpatient clinic regularly and were scheduled to undergo postoperative echocardiography between 1 and 12 months later. The index date was defined as the day of the post-op echocardiography. The primary outcomes were cardiovascular-related death, heart failure hospitalization, redone TV surgery, and heart transplantation. Cardiovascular-related death was defined as death from myocardial infarction, aggravation of heart failure, sudden cardiac death, or ischemic stroke. Heart failure hospitalization was defined as symptoms of dyspnea with a New York Heart Association (NYHA) grade of at least 3 requiring medications such as vasodilators or diuretics, elevated N-terminal pro-brain natriuretic peptide, and pulmonary congestion or pleural effusion on a chest X-ray. If a patient had two or more clinical events, only the first event was included as an outcome. The surgical mortality was defined as any cause of death within 30 days after surgery or before hospital discharge. We carefully reviewed medical records to find those outcomes, and follow-up was ended on the last day of April 2021 or whenever a primary outcome event occurred.

### Echocardiography

Standard 2D and Doppler measurements were performed by using a standard commercially available ultrasound machine (Vivid E9 color Doppler ultrasound M5S probe; GE Medical Systems, Chicago, IL; Philips iE33 color Doppler ultrasound X5-1 and S8-3 probe; Philips Healthcare, Netherlands) with a 2.5–3.5 MHz, in accordance with the guidelines of the American Society of Echocardiography (12). To better categorize TR severity, we used a newly proposed grading method that divides severe TR into “severe,” “massive,” and “torrential” TR (13). Measurements of the RV end-diastolic area (EDA) and end-systolic area (ESA) were obtained from the apical 4-chamber focused RV view at the end-diastolic and end-systolic phases, respectively, and the RV fractional area change (FAC) was calculated as the ratio of the RV EDA and ESA. The vena contracta width was measured at the narrowest portion of the regurgitant jet from the apical 4-chamber focused RV view. The LVEF was calculated by the biplane Simpson’s method in the apical 4-chamber and 2-chamber views. The LV end-diastolic dimension (EDD) and end-systolic dimension (ESD) were measured as the distance between the LV interventricular septum and posterior wall from the M-mode at the end-diastolic and end-systolic phases, respectively. Residual TR after TV surgery was defined as at least moderate TR on the post-op echocardiogram.

### Speckle-Tracking Echocardiography

**FIGURE 2:**
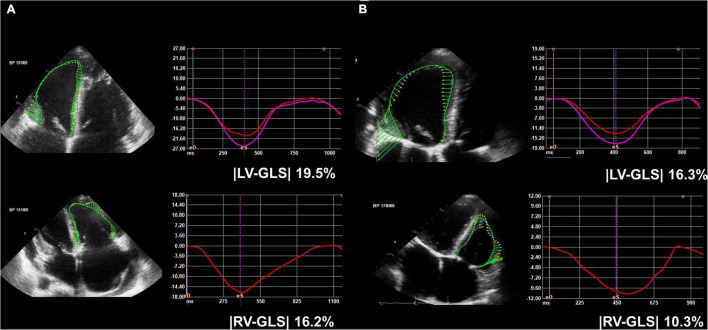
Representative figure of **(A)** pre-op and **(B)** post-op LV-GLS and RV-GLS measurements. LV-GLS was calculated from three standard apical images, and RV-GLS was calculated from RV focused apical echocardiographic images. LV, left ventricle; RV, right ventricle; GLS, global longitudinal strain; |LV-GLS|, absolute value of LV-GLS; |RV-GLS|, absolute value of RV-GLS.

We used the best images from three apical four-, three-, and two-chamber views and the RV-focused apical four-chamber view from both pre- and post-op echocardiographic data for our LV, RV, biventricular, and left atrial (LA) mechanical functional analyses. Those images were stored and exported to an offline storage device, and speckle-tracking echocardiography was performed using a vendor-independent software package (TomTec 2D cardiac performance analysis; Image Arena version 4.6, Munich, Germany). LV- and RV-GLS was measured according to the strain assessment guidelines by expert cardiologists who were blinded to clinical data (14–16). To measure LV-GLS, the LV endocardial borders were traced in apical four-, three-, and two-chamber views at the both the end-diastolic and end-systolic frames, and the TomTec software tracked the speckle on the three LV endocardial borders during a whole cardiac cycle (Figure 2). |LV-GLS| was defined as the absolute value of LV-GLS. To measure RV-GLS, the RV endocardial border, which included both the RV free wall and interventricular septum, was traced in the RV-focused apical four-chamber view at both the end-diastolic and end-systolic frames, and the TomTec software tracked the speckle on the RV endocardial borders during a whole cardiac cycle. |RV-GLS| was defined as the absolute value of RV-GLS. RV-free wall longitudinal strain (FWS) was defined as the strain value at the RV free wall. |RV-FWS| was defined as the absolute value of RV-FWS. Biventricular GLS was defined as the sum of LV-GLS and RV-FWS. Biventricular |GLS| was defined as the absolute value of biventricular GLS. To measure LA longitudinal strain, the LA endocardial border was traced in apical four and two-chamber views across the LA appendage and pulmonary veins. Then, the LA longitudinal strain curve through the whole cardiac cycle was analyzed by tracking the speckle on the LA endocardial borders. We selected 20 patients from the study cohort and analyzed the intra- and inter-observer reproducibility of the LV-GLS and RV-GLS measurements using a Bland-Altman analysis. The intra- and inter-class correlation coefficients of |LV-GLS| were 0.957 and 0.962, and those of |RV-GLS| were 0.989 and 0.989, respectively. The Bland-Altman analysis showed the limits of agreement (LOA) across a broad range of |LV-GLS| and |RV-GLS| values; the bias for intra- and inter-observer measurements of |LV-GLS| was 0.39% (range: −0.17 to 0.95%, 95% LOA) and 0.45% (range: 0.07–0.96%), and that of |RV-GLS| was 0.49% (range: −0.07 to 1.04%) and 0.17% (range: −0.39 to 0.73%), respectively.

### Statistical Analysis

Continuous variables are reported as the mean ± standard deviation, and categorical variables are reported as the frequency and percentage. Comparisons of baseline clinical, laboratory and echocardiographic parameters between the two groups (experiencing a primary outcome and not) were performed using Student’s *t*-test for continuous data and χ^2^ or Fisher’s exact test for categorical data. The predictive values of LV-, RV-, and biventricular GLS for the primary outcomes were calculated using a receiver operating characteristics (ROC) analysis. Kaplan-Meier survival analyses and log-rank tests were used to compare the clinical outcomes according to cutoff values for RV- and biventricular GLS during the follow-up period. Predictors of the primary outcomes were analyzed using multivariate nested Cox regression models. EuroSCORE II as a composite of demographic variables and echocardiographic parameters as pre- and post-op GLS of RV and biventricle were analyzed in the multivariate models. EuroSCORE II was included in every four models. Next, pre- and post-op RV-GLS were included in the first and second models. Pre- and post-op biventricular GLS were included in the third and fourth models. Differences were deemed significant at *P*-value < 0.05. All statistical analyses were performed using the SPSS software version 25.0 (IBM corporation, Armonk, NY).

## Results

### Baseline Characteristics

During a mean 3.9 ± 3.8 years after the post-op echocardiography, 24 (21.6%) patients experienced one of the primary outcomes. Among them, 5 died from cardiovascular causes, 17 had heart failure hospitalization, 1 had their TV surgery redone, and 1 had a heart transplant. The overall surgical mortality rate was 2.7% (*n* = 3). All of these patients died due to postoperative septic shock before the discharge. The surgery profile, baseline characteristics, medications, and laboratory results of patients with and without the primary outcomes are shown in Table 1. Of the total 111 patients, 89 (80.2%) underwent TV repair and 25 (22.5%) had concomitant MAZE operations. In patients with a primary outcome, a previous history of diabetes mellitus, chronic kidney disease, atrial fibrillation, or coronary artery disease was more common than in those without a primary outcome. NYHA class of III and IV were present over half (53.1%) of all patients. In terms of medication, patients with primary outcomes had taken more digoxin, anticoagulants, and diuretics than those without. In the laboratory findings, patients with primary outcomes had a lower level of albumin (3.8 ± 0.7 vs. 4.1 ± 0.5 mg/dL, *p* = 0.044) and a higher level of creatinine (1.1 ± 0.5 vs. 0.8 ± 0.2 mg/dL, *p* = 0.013) than those without primary outcomes. The EuroSCORE II was significantly higher in patients with primary outcomes than the controlled group (3.8 ± 2.3 vs. 1.9 ± 1.1%, *p* < 0.001).

**TABLE 1:** Baseline characteristics.

	Total (*n* = 111)	With outcome (*n* = 24)	Without outcome (*n* = 87)	*P*-value
Age, years	63.9 ± 12.8	67.2 ± 15.1	63.0 ± 12.1	0.162
Female sex, n (%)	76 (68.5)	15 (62.5)	61 (70.1)	0.477
BMI, kg/m^2^	23.6 ± 3.3	23.9 ± 3.7	23.5 ± 3.2	0.611
Hypertension, n (%)	73 (65.8)	18 (75.0)	55 (63.2)	0.282
Diabetes mellitus, n (%)	19 (17.1)	8 (33.3)	11 (12.6)	0.017
Dyslipidemia, n (%)	26 (23.4)	5 (20.8)	21 (24.1)	0.735
CKD, n (%)	11 (9.9)	5 (20.8)	6 (6.9)	0.043
Atrial fibrillation, n (%)	86 (77.5)	24 (100.0)	62 (71.3)	0.003
CAD, n (%)	7 (6.3)	4 (16.7)	3 (3.4)	0.018
Chronic lung disease, n (%)	18 (16.2)	5 (20.8)	13 (14.9)	0.488
Etiology of TR				0.349
Primary, n (%)	10 (9.0)	1 (4.2)	9 (10.3)	
Secondary, n (%)	101 (91.0)	23 (95.8)	78 (89.7)	
TV surgery profile				
TV replacement, n (%)	22 (19.8)	6 (25.0)	16 (18.4)	0.472
TV repair, n (%)	89 (80.2)	18 (75.0)	71 (81.6)	
Concomitant MAZE, n (%)	25 (22.5)	4 (16.7)	21 (24.1)	0.438
NYHA class				0.149
II, n (%)	52 (46.8)	8 (33.3)	44 (50.6)	
III, n (%)	40 (36.0)	9 (37.5)	31 (35.6)	
IV, n (%)	19 (17.1)	7 (29.2)	12 (13.8)	
**Medications, n (%)**				
RAAS blockers	56 (50.5)	12 (50.0)	44 (50.6)	0.960
Beta-blockers	40 (36.0)	6 (25.0)	34 (39.1)	0.203
CCB	18 (16.2)	7 (29.2)	11 (12.6)	0.052
Digoxin	35 (31.5)	12 (50.0)	23 (26.4)	0.028
Statin	35 (31.5)	9 (37.5)	26 (29.9)	0.477
Antiplatelets	19 (17.1)	5 (20.8)	14 (16.1)	0.585
Anticoagulants	76 (68.5)	21 (87.5)	55 (63.2)	0.023
Diuretics	96 (86.5)	24 (100.0)	72 (82.8)	0.029
Loop diuretics	84 (75.7)	20 (83.3)	64 (73.6)	0.323
Mineralocorticoid receptor antagonist	53 (47.7)	13 (54.2)	40 (46.0)	0.477
**Laboratory findings**				
Hemoglobin, g/dL	12.5 ± 2.2	11.7 ± 2.8	12.8 ± 1.9	0.086
Platelet, × 10^6^ mL	196.2 ± 83.4	183.0 ± 98.5	199.9 ± 78.9	0.384
Total protein, mg/dL	6.8 ± 0.8	6.6 ± 1.1	6.9 ± 0.7	0.376
Albumin, mg/dL	4.0 ± 0.5	3.8 ± 0.7	4.1 ± 0.5	0.044
Creatinine, mg/dL	0.9 ± 0.3	1.1 ± 0.5	0.8 ± 0.2	0.013
Total bilirubin, mg/dL	0.8 ± 0.4	0.9 ± 0.6	0.8 ± 0.4	0.190
INR	1.17 ± 0.4	1.25 ± 0.4	1.15 ± 0.4	0.159
EuroSCORE II, %	2.3 ± 1.6	3.8 ± 2.3	1.9 ± 1.1	< 0.001

*BMI, body mass index; CKD, chronic kidney disease; CAD, coronary artery disease; TR, tricuspid regurgitation; NYHA, New York Heart Association; RAAS, renin-angiotensin-aldosterone system; CCB, calcium channel blockers; INR, international normalized ratio.*

The mean period between preoperative echocardiography and surgery was 1.0 ± 1.2 months and the mean period between surgery and postoperative echocardiography was 7.2 ± 4.4 months. The preoperative and postoperative echocardiographic parameters are presented in Table 2. In the preoperative echocardiography, 13 of 111 (11.7%) patients were graded with “massive” TR. Patients with primary outcomes had more-dilated LV chambers, lower RV FAC, |RV-GLS|, |RV-FWS|, and biventricular |GLS| than those without primary outcomes. In the postoperative echocardiography, residual TR was reported in 29 (26.1%) patients. Patients with primary outcomes had larger LA and lower RV FAC, |RV-GLS|, |RV-FWS|, |LV-GLS|, biventricular |GLS|, and LA reservoir strain than those without primary outcomes. Changes in strain parameters of the total study population are shown in Supplementary Figure 1. |RV-GLS|, |RV-FWS|, and biventricular |GLS| revealed significant decreases after TV surgery.

**TABLE 2:** Echocardiographic characteristics.

	Total (*n* = 111)	With outcome (*n* = 24)	Without outcome (*n* = 87)	*P*-value
***Pre-op TTE***				
TR grade				0.194
Severe, n (%)	98 (88.3)	23 (95.8)	75 (86.2)	
Massive, n (%)	13 (11.7)	1 (2.8)	12 (13.8)	
Torrential, n (%)	0 (0.0)	0 (0.0)	0 (0.0)	
RV S′, cm/s	10.6 ± 3.1	9.3 ± 2.8	11.0 ± 3.1	0.023
RV FAC, %	37.7 ± 10.2	31.7 ± 10.5	39.4 ± 9.6	0.001
|RV-GLS|, %	20.7 ± 6.5	16.0 ± 6.2	22.0 ± 6.0	<0.001
|RV-FWS|, %	21.3 ± 8.1	17.0 ± 7.4	22.5 ± 7.9	0.003
RV EDA, mm	27.5 ± 10.5	26.4 ± 11.3	27.9 ± 10.3	0.537
RV ESA, mm^2^	16.8 ± 6.4	17.9 ± 8.1	16.6 ± 5.8	0.375
VC Width, mm	9.7 ± 3.0	9.8 ± 2.1	9.7 ± 3.2	0.791
RVSP, mmHg	42.4 ± 11.2	46.0 ± 15.0	41.5 ± 9.8	0.170
LV EF, %	61.7 ± 8.6	60.2 ± 9.4	62.2 ± 8.3	0.325
|LV-GLS|, %	18.7 ± 3.9	18.0 ± 4.7	18.9 ± 3.6	0.351
LV EDD, mm	47.5 ± 6.9	50.0 ± 7.7	46.8 ± 6.5	0.043
LV ESD, mm	32.6 ± 5.8	34.8 ± 6.7	32.1 ± 5.4	0.039
Biventricular |GLS|, %	40.0 ± 10.1	34.9 ± 10.7	41.4 ± 9.6	0.006
LAVI, mL/m^2^	65.0 ± 47.4	74.5 ± 40.6	62.4 ± 49.0	0.271
E/e’	11.4 ± 6.7	13.6 ± 6.6	10.9 ± 6.7	0.126
LA reservoir strain, %	17.4 ± 9.4	16.1 ± 8.3	17.8 ± 9.7	0.434
***Post-op TTE***				
Residual TR	29 (26.1)	9 (37.5)	20 (23.0)	0.152
RV FAC, %	32.2 ± 8.7	24.8 ± 9.7	34.2 ± 7.2	<0.001
|RV-GLS|, %	18.3 ± 5.2	13.8 ± 5.3	19.5 ± 4.4	<0.001
|RV-FWS|, %	18.7 ± 5.8	14.4 ± 6.2	19.9 ± 5.1	<0.001
RV EDA, mm^2^	21.2 ± 6.5	22.8 ± 8.1	20.8 ± 5.9	0.273
RV ESA, mm^2^	14.5 ± 5.1	17.2 ± 6.7	13.7 ± 4.4	0.020
VC Width, mm	2.9 ± 2.6	3.9 ± 3.3	2.7 ± 2.3	0.105
LV EF, %	64.1 ± 8.2	60.9 ± 11.7	65.0 ± 6.8	0.114
|LV-GLS|, %	18.4 ± 4.2	16.4 ± 5.1	19.0 ± 3.7	0.024
LV EDD, mm	49.1 ± 5.6	50.0 ± 7.2	48.9 ± 5.1	0.476
LV ESD, mm	33.2 ± 5.5	34.9 ± 7.8	32.8 ± 4.5	0.212
|Biventricular GLS|, %	37.1 ± 8.8	30.7 ± 10.6	38.9 ± 7.5	<0.001
LAVI, mL/m^2^	61.0 ± 33.3	73.5 ± 35.5	57.5 ± 32.0	0.036
E/e’	16.2 ± 7.2	18.0 ± 4.5	15.8 ± 7.6	0.298
LA reservoir strain, %	15.5 ± 10.4	8.6 ± 7.0	17.4 ± 10.4	<0.001

*RV, right ventricle; FAC, fractional area change; S′, systolic excursion velocity; |GLS|, absolute value of global longitudinal strain; |FWS|, absolute value of free wall strain; EDA, end diastolic area; ESA, end systolic area; VC, vena contracta; HV, hepatic vein; EF, ejection fraction; EDD, end diastolic dimension; ESD, end systolic dimension; LAVI, left atrial volume index; E/e′, ratio of early diastolic mitral inflow velocity to early diastolic mitral annular tissue velocity.*

### Predictive Value of Global Longitudinal Strain for Primary Outcomes

**FIGURE 3:**
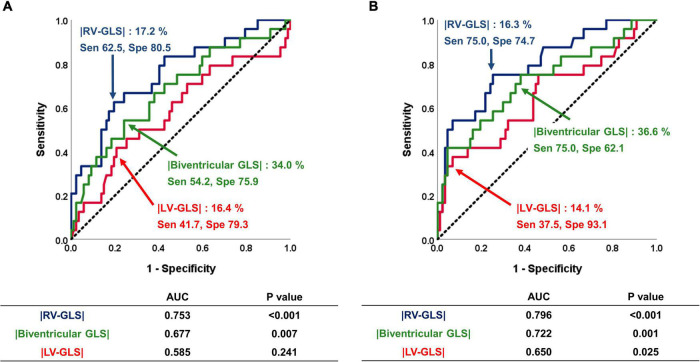
Predictive value of |LV-GLS|, |RV-GLS|, and biventricular |GLS| for the occurrence of negative clinical outcomes. **(A)** On the pre-op echocardiogram, |RV-GLS| and biventricular |GLS| had significant predictive value for clinical outcomes. **(B)** On the post-op echocardiogram, |RV-GLS| showed better predictive value for clinical outcomes than biventricular |GLS| and |LV-GLS|. LV, left ventricle; |GLS|, absolute value of global longitudinal strain; RV, right ventricle.

The ROC analysis for the predictive value of RV-, biventricular, and LV-GLS for the primary outcomes is shown in Figure 3. Both pre- and post- echocardiographic strain values showed that |RV-GLS| and biventricular |GLS| had significant predictive value for the primary outcomes. Among the three different GLS values, RV-GLS showed the largest area under the curve for predicting outcomes. The cut-off values of preoperative |RV-GLS| and postoperative |RV-GLS| were 17.2 and 16.3%, respectively, and the cut-off values of pre- and postoperative biventricular |GLS| were 34.0 and 36.6%, respectively. All of those values show acceptable sensitivity and specificity, as shown in Figure 3. Interestingly, the preoperative LV-GLS value did not predict the outcomes. Only a postoperative |LV-GLS| lower than 14.1% predicted the outcomes with high specificity.

**FIGURE 4:**
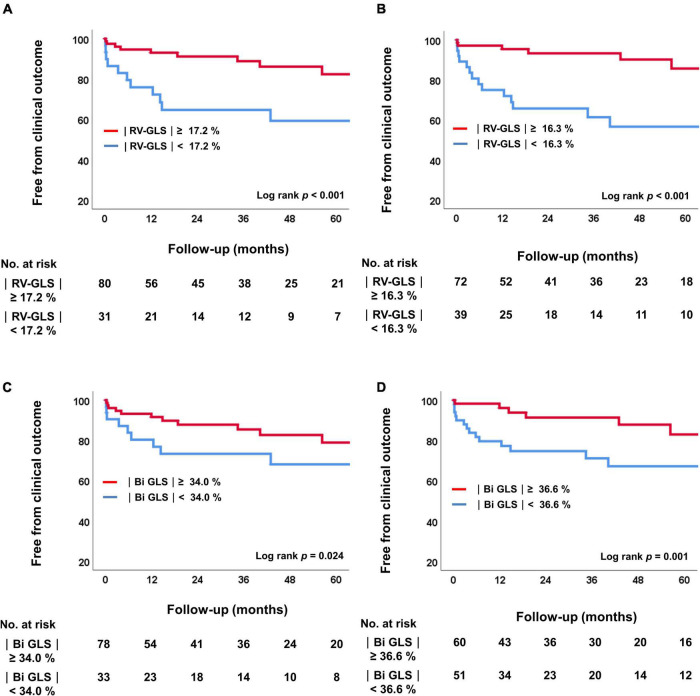
Kaplan-Meier analysis of freedom from clinical outcomes. **(A)** Comparison of two groups according to the cutoff value for |RV-GLS|_*preop*_. **(B)** Comparison of four groups according to the cutoff value for |RV-GLS|_*postop*_. **(C)** Comparison of two groups according to the cutoff value for biventricular |GLS|_*preop*_. **(D)** Comparison of four groups according to the cutoff value for biventricular |GLS|_*postop*_. RV, right ventricle; |GLS|, absolute value of global longitudinal strain.

Figure 4 shows the Kaplan-Meier survival curves for groups divided by the cut-off values of |RV-GLS| and biventricular |GLS| in both the preoperative and postoperative studies. Regardless of the time point, biventricular mechanical dysfunction, including RV dysfunction, correlated with significant differences in the prognosis of these patients (log rank *p* = 0.024 in preoperative study, log rank *p* = 0.001 in postoperative study). Based on the cut-off values of preoperative |RV-GLS| and |LV-GLS|, we divided the study patients into 4 groups according to whether the RV and LV strain values were preserved and performed another Kaplan-Meier analysis for clinical outcomes (Supplementary Figure 2). In the results, patients who had reduced both RV and LV strain on preoperative echocardiography had the worst clinical outcomes (*p* = 0.003).

In the multivariate nested Cox regression models, higher EuroSCORE II was an independent predictor of the primary outcomes in every model. When preoperative |RV-GLS| and postoperative |RV-GLS| were added sequentially in model 1 and model 2, lower preoperative |RV-GLS| [hazard ratio (HR) 0.89, confidence interval (CI) 0.82–0.96, *p* = 0.006] and postoperative |RV-GLS| (HR 0.84, CI 0.78–0.91, *p* < 0.001) were significant independent predictors of the primary outcomes. In the same way, when pre- and postoperative biventricular |GLS| were added sequentially in model 3 and model 4, preoperative biventricular |GLS| was not independently related to primary outcomes (HR 0.97, CI 0.93–1.01, *p* = 0.111). However, lower postoperative biventricular |GLS| (HR 0.93, CI 0.89–0.98, *p* = 0.005) were significant independent predictors of the primary outcomes in model 4 (Table 3).

**TABLE 3:** Multivariable Cox regression models for clinical outcomes.

	Model 1			Model 2		
		
	HR	95% CI	*P*-value	HR	95% CI	*P*-value
EuroSCORE II	1.56	1.30–1.88	<0.001	1.59	1.32–1.92	<0.001
Preoperative |RV-GLS|	0.89	0.82–0.96	0.003			
Postoperative |RV-GLS|				0.84	0.78–0.91	<0.001

	**Model 3**			**Model 4**		
		
	**HR**	**95% CI**	***P*-value**	**HR**	**95% CI**	***P*-value**

EuroSCORE II	1.64	1.37–1.97	<0.001	1.56	1.30–1.89	<0.001
Preoperative |biventricular GLS|	0.97	0.93–1.01	0.118			
Postoperative |biventricular GLS|				0.93	0.89–0.98	0.005

*Model 1: EuroSCORE II + Preoperative |RV-GLS|.*

*Model 2: EuroSCORE II + Postoperative |RV-GLS|.*

*Model 3: EuroSCORE II + Preoperative |biventricular GLS|.*

*Model 4: EuroSCORE II + Postoperative |biventricular GLS|.*

*RV, right ventricle; |GLS|, absolute value of global longitudinal strain.*

### Incremental Prognostic Value of Right Ventricular and Biventricular Global Longitudinal Strain

**FIGURE 5:**
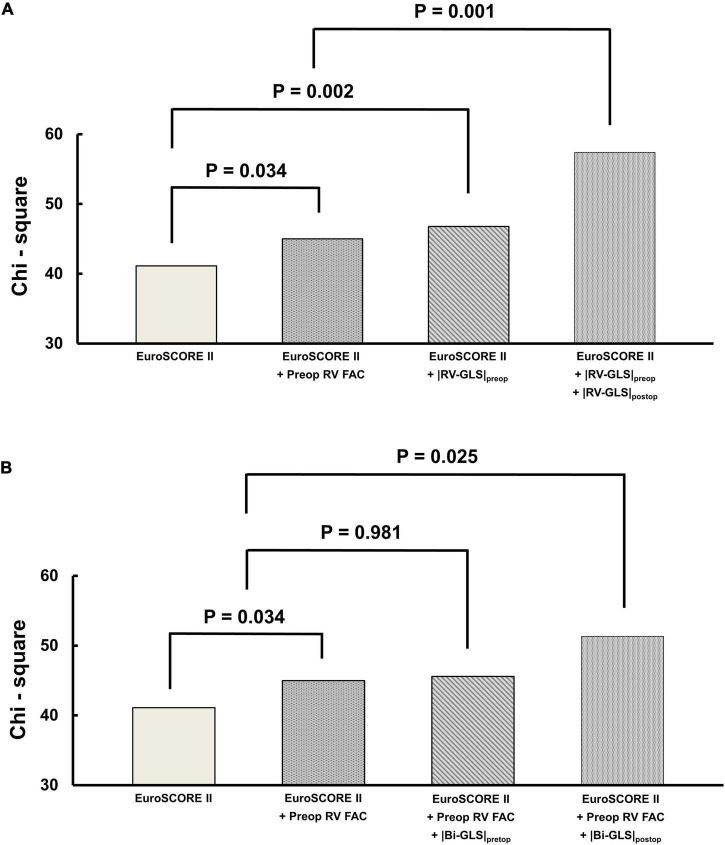
Incremental prognostic value of preoperative and postoperative **(A)** |RV-GLS| and **(B)** biventricular |GLS| over EuroSCORE II. RV, right ventricle; FAC, fractional area change; |GLS|, absolute value of global longitudinal strain.

The incremental prognostic values of RV and biventricular GLS are shown in Figure 5. In the model that included |RV-GLS| for prognosis (Figure 5A), the addition of preoperative and postoperative |RV-GLS| to EuroSCORE II significantly improved the model’s predictive value for the primary outcomes (*p* = 0.002, *p* = 0.001, respectively) and the preoperative |RV-GLS| had more sensitive predictive value than preoperative FAC (*p* = 0.032). In the model that included biventricular |GLS| for prognosis with EuroSCORE II (Figure 5B), preoperative biventricular |GLS| did not improve the predictive value (*p* = 0.981), but postoperative biventricular |GLS| did (*p* = 0.025).

## Discussion

The principal findings of this study are as follows: (1) Clinical events after isolated TV surgery are common (21.6%), including cardiovascular deaths in 4.5% of patients during a mean 3.9 years. (2) The preoperative GLS value that best predicted the outcomes after isolated TV surgery was |RV-GLS|, which predicted a poor prognosis when it was 17.2% or less. This finding suggests that |RV-GLS| has value in identifying high risk for poorer outcomes after isolated TV surgery. (3) Biventricular GLS, which comprehensively evaluated the mechanical function of both ventricles, also showed predictive value, particularly in the postoperative evaluation. Overall, our findings imply that a GLS evaluation could be used to stratify the risk of post-surgical prognosis of patients with severe isolated TR that could be masking intrinsic ventricular dysfunction.

### Right Ventricular and Left Ventricular Functional Changes in Patients With Severe Isolated Tricuspid Regurgitation

As isolated TR increases in severity, RV volume overload proceeds, and eventually chronic remodeling of the right chambers occurs. Although those phenomena cause intrinsic RV dysfunction due to stress on the RV myocardium, that RV dysfunction is often masked to some extent due to the volume overload in patients with severe TR (17). Severe isolated TR can also affect the LV chamber. RV overload and consequent RV dysfunction interfere with LV filling by RV dilation and leftward shifting of the interventricular septum by ventricular interdependence. Furthermore, that causes decreased LV distensibility and elastance (6). Therefore, severe TR is often accompanied by LV dysfunction and low stroke volume. In this physiology of underfilled LV, it has limitations on its own function of elastic coil and re-coil of LV myocardial fiber. Therefore, its function might be concealed unless the correction with TV intervention. However, after the correction of severe TR, LV filling is restored, which can unmask left-side heart problems.

With that theoretical background on the change of biventricular function in severe TR, we hypothesized in this study that GLS in both ventricles, which measures the myocardial mechanical function less volume dependently than other tests, would be important in predicting the prognoses of patients with severe isolated TR as they planned surgery. The current guideline does not provide definite timing for TV surgery because of limitations in clinical data, especially in isolated TR, which generally has indolent progression (3, 4). Although severe TR is a strong predictor of poor clinical outcomes, some outcome data from isolated TV surgery have shown poor results, with in-hospital mortality as high as 8–10% (2, 18). Those results are thought to result from the lack of understanding and standards for timing the intervention. In our study results, in-hospital mortality was reported as 2.7% and the results seemed very low. However, considering the 2 patients who dropped out from the strict study inclusion criteria, which excluded the patients who had the clinical events before the postoperative echocardiographic data after at least 1 month of TV surgery, the actual in-hospital mortality was 4.4%. In another previous study that demonstrated the prognostic value of |RV-FWS| in patients who underwent isolated TV surgery (19), the surgical mortality was reported as 5.2% and this result was comparable with our study. That was because of the similarity with the strict cohort criteria of the study, which excluded patients with significant left-sided valve disease, reduced LV systolic function, and primary pulmonary hypertension. With the development of alternative interventional treatments such as trans catheter edge-to-edge repair (20, 21), there is an increasing need to define a reference point for isolated TV surgery.

Therefore, interest in determining optimal surgery timing and analyzing prognostic factors has increased. In 2020, Dreyfus et al. identified the determinants of outcomes after isolated TV surgery in 5,661 patients from French tertiary centers (22). They reported that NYHA class III/IV, at least moderate RV dysfunction, and lower prothrombin time were independent determinants of clinical outcomes. However, they did not quantitatively assess RV function. Another previous study investigated the preoperative predictors of clinical outcomes after isolated TV surgery in 238 patients (23) and found that the TR jet area (≥30 cm^2^), RA pressure (≥15 mmHg), age, and hemoglobin level were independent predictors of clinical outcomes. Although those factors were calculated by quantitative methods, they have limitations in reflecting RV function. Our study demonstrates the prognostic value of GLS measurements. RV-GLS is a better tool for assessing RV function in isolated TR than conventional echocardiography, which has limitations due to the asymmetrical RV geometry, volume dependency, and difficulty in defining the true endocardial border because of heavy trabeculation (24). In assessing LV function, LV-GLS might be more accurate and sensitive than LVEF under the condition of LV under-filling in severe TR or re-filling after TV surgery (25). There was a similar previous study that performed the analysis of LV function in a group of patients with ventricular interdependence. In the study, LV myocardial function by LV-GLS was evaluated in 54 patients with pulmonary hypertension and 54 control subject (26). The study revealed that patients with pulmonary hypertension had a reduced value of LV-GLS (−18.8 vs. −20.0%, *p* = 0.005) than matched controls, although all the groups had a normal range of LV EF. It showed that LV-GLS reflected the impaired LV function more accurately than LV EF in patients with ventricular independence. In this study, preoperative |RV-GLS| was the strongest indicator, with a cut-off point of 17.2%, and it would be worth considering as a useful indicator in identifying the risk of post-operative outcomes after TV surgery in other population of isolated TR. On the other hand, preoperative LV-GLS did not meaningfully predict the prognosis of patients after TV surgery. Although biventricular GLS was significantly associated with clinical outcomes, we attribute that to the significance of RV-GLS.

### Biventricular Global Longitudinal Strain in Patients With Severe Isolated Tricuspid Regurgitation

Because isolated TR affects not only the RV but also the LV, integrative functional measurements of both ventricles are needed. We devised an indicator called *biventricular GLS* to comprehensively evaluate the function of both ventricles. It is a combination of RV-FWS and LV-GLS to reflect the functioning of the RV and LV together and can be used to determine the severity of isolated TR. A previous study demonstrated the prognostic value of |RV-FWS| in 115 patients who received isolated TV surgery in 2 tertiary centers between 2005 and 2019 (19). It showed that |RV-FWS| below 24% in pre-op echocardiography was associated with the primary endpoint. Our results in this study are consistent with those in indicating that RV strain is an important imaging prognosticator in patients undergoing isolated TV surgery. However, we are the first to identify the post-surgical prognostic implications of biventricular GLS in patients with severe isolated TR. There have been no other studies that comprehensively assessed the function of both ventricles by summation of GLS, but this trial is thought to provide a considerable foundation for clinical trials related to the prognostic evaluation of other clinical disease spectrums affecting the function of both ventricles and analysis of disease severity. Also, 62.6% of patients in the previous study had had previous left-side valve surgery, whereas we excluded patients who underwent previous left-side valve surgery or open heart surgery from this study. That is, our study minimized confounding by LV mechanical dysfunction, so we provide clinically important information about the optimal timing for TR intervention and outcome prediction in operation-naïve patients with severe isolated TR. We found that biventricular |GLS| shared a predictive index with |RV-GLS|, and both metrics showed significant correlations before and after surgery. However, both before and after surgery, |RV-GLS| showed higher predictive power than biventricular |GLS|. Thus, we suggest that RV function plays a more important role than LV function in the prognosis of patients after isolated TV surgery.

### Limitations

This study has several limitations. First, this study was designed retrospectively for patients who visited regularly. Intervals of follow-up echocardiography after isolated TV surgery were not consistent in all patients. However, we tried to set the period between the surgery and follow-up echocardiography as consistently as possible to minimize the effects of those limitations. More comprehensive prospective multicenter studies of patients with isolated TR are needed. Second, the entire study cohort was relatively small (*n* = 111) due to the incidence of isolated TV surgery. However, we selected the patients with isolated TR using strict inclusion and exclusion criteria to identify the exact effects of factors after isolated TV surgery. Third, the echocardiographic parameters of RV and LV strain might be inconsistent because the echocardiography for each patient was not performed on the same equipment. However, we used vendor-independent software to minimize the measurement errors of our expert operators. Fourth, not all the patients did not perform right heart catheterization so the hemodynamic data such as right-sided intracardiac pressures and the cardiac index of the entire study cohorts could not be assessed. Fifth, two patients who had a clinical event from the time of surgery until follow-up echocardiography were excluded and this might introduce selection bias in terms of assessing postoperative prognosis.

## Conclusion

In patients with severe isolated TR undergoing TV surgery, |RV-GLS| under 17.2% is closely associated with a poor prognosis, and biventricular |GLS| under 34.0%, mainly depending on the |RV-GLS|, is related to the poor prognosis. Therefore, assessing |RV-GLS| and biventricular |GLS| by speckle-tracking echocardiography could have benefits in identifying high risk of poorer outcomes after TV surgery. Further prospective multicenter studies with better adjustment are warranted to establish the risk stratification of isolated TV surgery.

## Data Availability Statement

The original contributions presented in this study are included in the article/Supplementary Material, further inquiries can be directed to the corresponding author/s.

## Ethics Statement

The studies involving human participants were reviewed and approved by the Institutional Review Board of Yonsei University Health System (IRB number: 4-2021-0929). Written informed consent for participation was not required for this study in accordance with the national legislation and the institutional requirements.

## Author Contributions

D-YK and CS contributed to the concept and design of this study. JS, IC, SHL, and SL contributed to acquisition, analysis, and interpretation of the data. G-RH and J-WH contributed to drafting of the manuscript and statistical analysis. D-YK, J-WH, and CS contributed to revision and finalize the manuscript. All authors contributed to the article and approved the submitted version.

## Conflict of Interest

The authors declare that the research was conducted in the absence of any commercial or financial relationships that could be construed as a potential conflict of interest.

## Publisher’s Note

All claims expressed in this article are solely those of the authors and do not necessarily represent those of their affiliated organizations, or those of the publisher, the editors and the reviewers. Any product that may be evaluated in this article, or claim that may be made by its manufacturer, is not guaranteed or endorsed by the publisher.
