# Supplementary material for: Prognostic Implications of Biventricular Global Longitudinal Strain in Patients With Severe Isolated Tricuspid Regurgitation
Source: Front Cardiovasc Med. 2022 Aug 3;9:908062. doi: 10.3389/fcvm.2022.908062 (PMC9381843; doi:10.3389/fcvm.2022.908062)
Supplement: Supplementary file 1 [file Data_Sheet_1.docx]

Supplementary Material


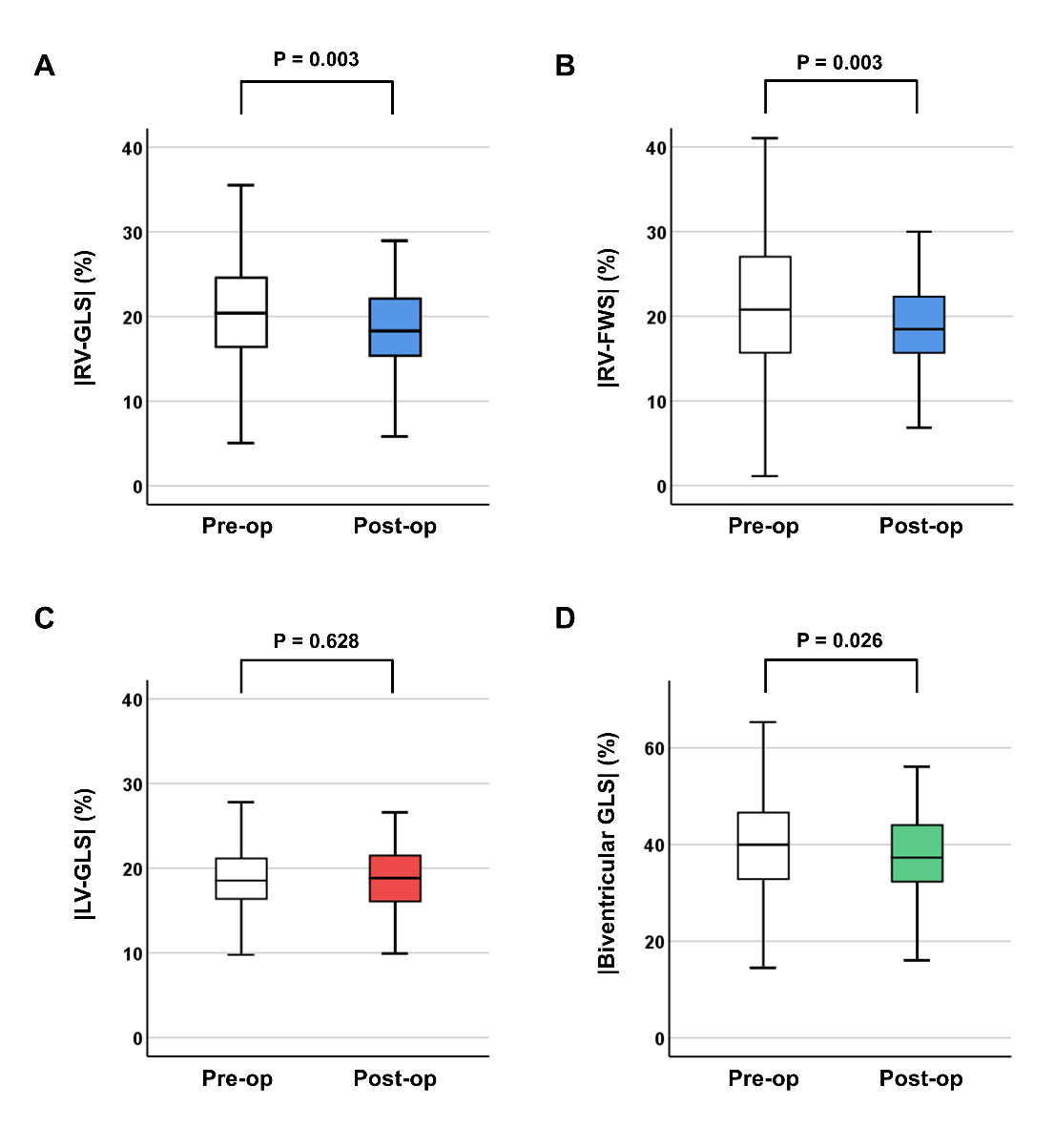


**Supplementary Figure 1.** The changes in |RV-GLS|, |RV-FWS|, |LV-GLS|, and biventricular |GLS| on pre-operative and post-operative echocardiogram. **(A)(B)** |RV-GLS| and |RV-FWS| decreased after TV surgery. **(C)** |LV-GLS| did not show the significant difference between pre-operative and post-operative echocardiogram. However, **(D)** biventricular |GLS|, which was defined as the sum of |LV-GLS| and |RV-FWS|, decreased after TV surgery. RV, right ventricle; |GLS|, absolute value of global longitudinal strain; |FWS|, absolute value of free wall strain; LV, left ventricle.


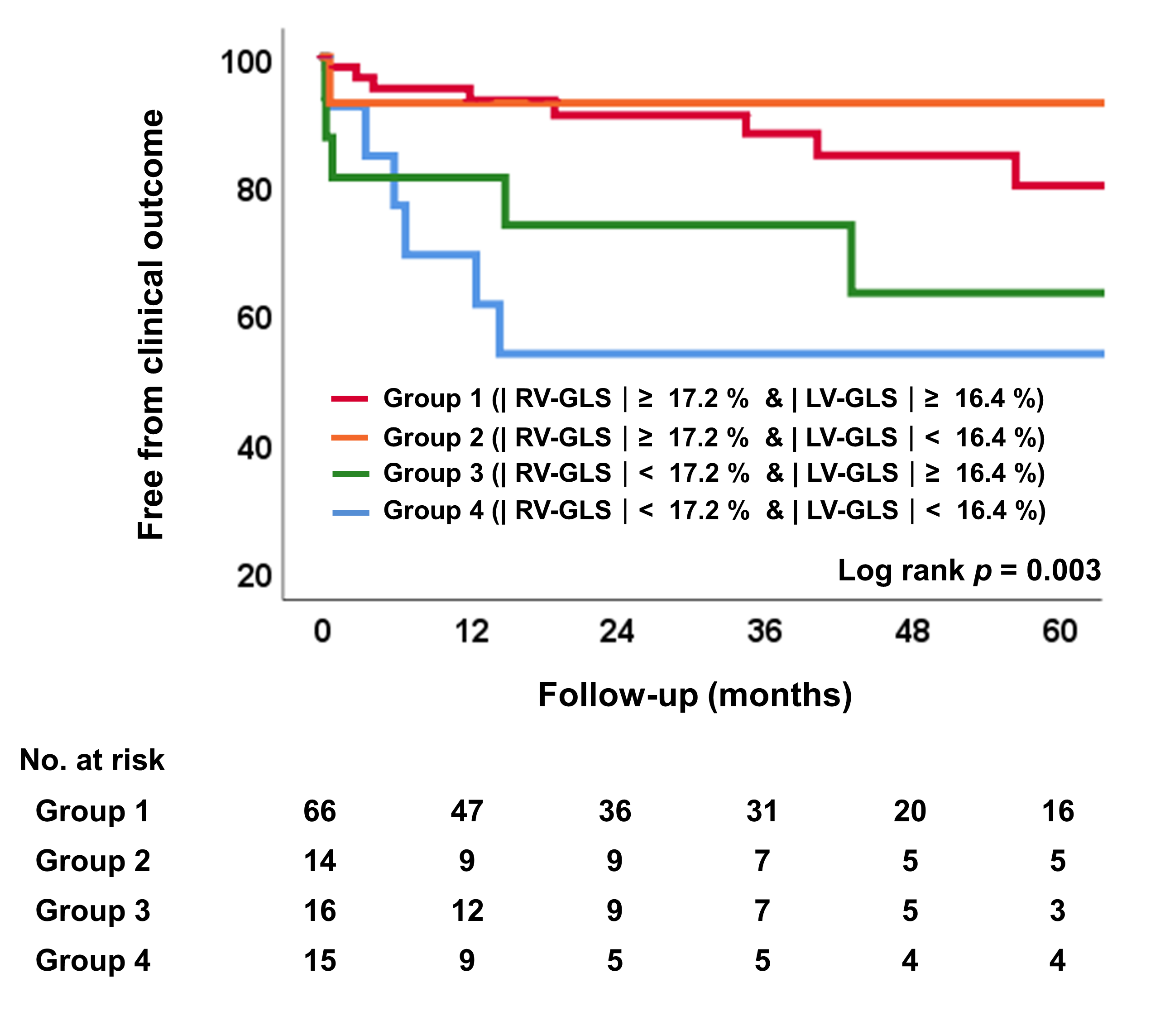


**Supplementary Figure 2.** Kaplan-Meier analysis of freedom from clinical outcomes among the patients who were divided into 4 groups according to the cut-off value of preoperative |RV-GLS| and |LV-GLS|. Group 4 (reduced both |RV-GLS| and |LV-GLS| values) had the worst clinical outcomes (p=0.003). |RV-GLS|, the absolute value of global longitudinal strain of right ventricle; |LV-GLS|, the absolute value of global longitudinal strain of left ventricle.
